# Characterization of the complete plastid genome of *Craniotome* Rchb., a monotypic genus of Pogostemoneae, Lamiaceae

**DOI:** 10.1080/23802359.2020.1793700

**Published:** 2020-07-23

**Authors:** Yongbin Wu, Gang Yao

**Affiliations:** College of Forestry and Landscape Architecture, South China Agricultural University, Guangzhou, China

**Keywords:** Lamiaceae, Pogostemoneae, plastid phylogenomics, *Craniotome*

## Abstract

*Craniotome* Rchb. is a monotypic genus of Lamiaceae. In this study, the complete plastid genome of the species *C. furcata* (Link) Kuntze was sequenced and assembled. The plastid genome obtained is 152,521 bp in length, including a pair of inverted repeat (IRa and IRb) regions of 25,596 bp, a large single-copy (LSC) region of 83,690 bp, and a small single-copy (SSC) region of 17,639 bp. The genome encoded 113 unique genes, including 79 protein-coding genes, 4 ribosomal RNA genes, and 30 transfer RNA genes. The overall GC content of the genome obtained is 38.29%. The phylogenetic analysis based on 37 plastid genome of Lamiaceae revealed that the genus *Craniotome* was sister to the *Anisomeles*-*Pogostemon* clade with strong support.

*Craniotome* Rchb. is a monotypic genus of Pogostemoneae, Lamiaceae. It is widely distributed from India, Nepal, Bhutan, to China and the Indo-China Peninsula (Li and Hedge 1994). The species *C. furcata* (Link) Kuntze is a perennial herb and grows usually in boscage, roadsides, at the elevation of 900‒3200 m (Li and Hedge 1994).

The fresh leaves of *C. furcata* were collected from Yongde Hsien, Yunnan province (China; N 24°12′, E 99°42′). Total geonomic DNA of the species was extracted using the modified CTAB method (Doyle and Doyle 1987). Voucher specimen (*G. Yao 346*) was deposited in the herbarium of South China Botanical Garden, Chinese Academy of Sciences (IBSC). The DNA extracted was sequenced using the Illumina HisSeq 2500 Sequencing System. The clean reads obtained from *C. furcata* were assembled using the software GetOrganelle (Jin et al. 2018). The assembled plastid genome was annotated with PGA (Qu et al. 2019) and then deposited in the GenBank (Accession number MT648820).

Structural analysis of the complete plastid genome of *C. furcata* exhibits a typical quadripartite circular structure with 152,521 bp in length, containing a pair of inverted repeat (IRa and IRb) regions of 25,596 bp, a large single-copy (LSC) region of 83,690 bp, and a small single-copy (SSC) region of 17,639 bp. The overall GC content of *C. furcata* plastid genomes is 38.29% (LSC, 36.41%; SSC, 32.37%; IR, 43.40%). The genome encoded 113 unique genes, including 79 protein-coding genes, 4 ribosomal RNA genes (*rrn4.5*, *rrn5*, *rrn16* and *rrn23*), and 30 transfer RNA genes. Seventeen genes, including six protein-coding genes (*ndhB*, *rpl2*, *rpl23*, *rps12*, *rps7* and *ycf2*), four ribosomal RNA gens, and seven transfer RNA genes (*trnA-UGC*, *trnI-CAU*, *trnI-GAU*, *trnL-CAA*, *trnN-GUU*, *trnR-ACG*, *trnV-GAC*) were found to be duplicated in IR regions. Sixteen genes (*atpF*, *ndhA*, *ndhB*, *petB*, *petD*, *rpl2*, *rpl16*, *rpoC1*, *rps12*, *rps16*, *trnA-UGC*, *trnG-UCC*, *trnI-GAU*, *trnK-UUU*, *trnL-UAA*, *trnV-UAC*) contained one intron and two genes (*clpP* and *ycf3*) contained two introns.

In this study, *C. furcata* and 36 published complete plastid genomes of Lamiaceae were used to construct a maximum likelihood (ML) phylogenetic tree. The species *Paulownia tomentose* Steud. from Paulowniaceae was selected as an outgroup, the phylogenetic framework of Lamiales provided by Liu et al. (2020) was referred. Phylogenetic results showed that all of the Pogostemoneae species formed a well-supported clade. The species *C. furcata* diverged firstly within the tribe Pogostemoneae and it was sister to the *Anisomeles*-*Pogostemon* clade with 100% bootstrap support value (Figure 1), which was in agreement with preciously published phylogenetic studies (Li et al. 2016; Yao et al. 2016). The *C. furcata* plastid genome obtained here provided new genomic data for exploring the phylogenetic relationship and evolutionary history of Lamiaceae members.

**Figure 1.**
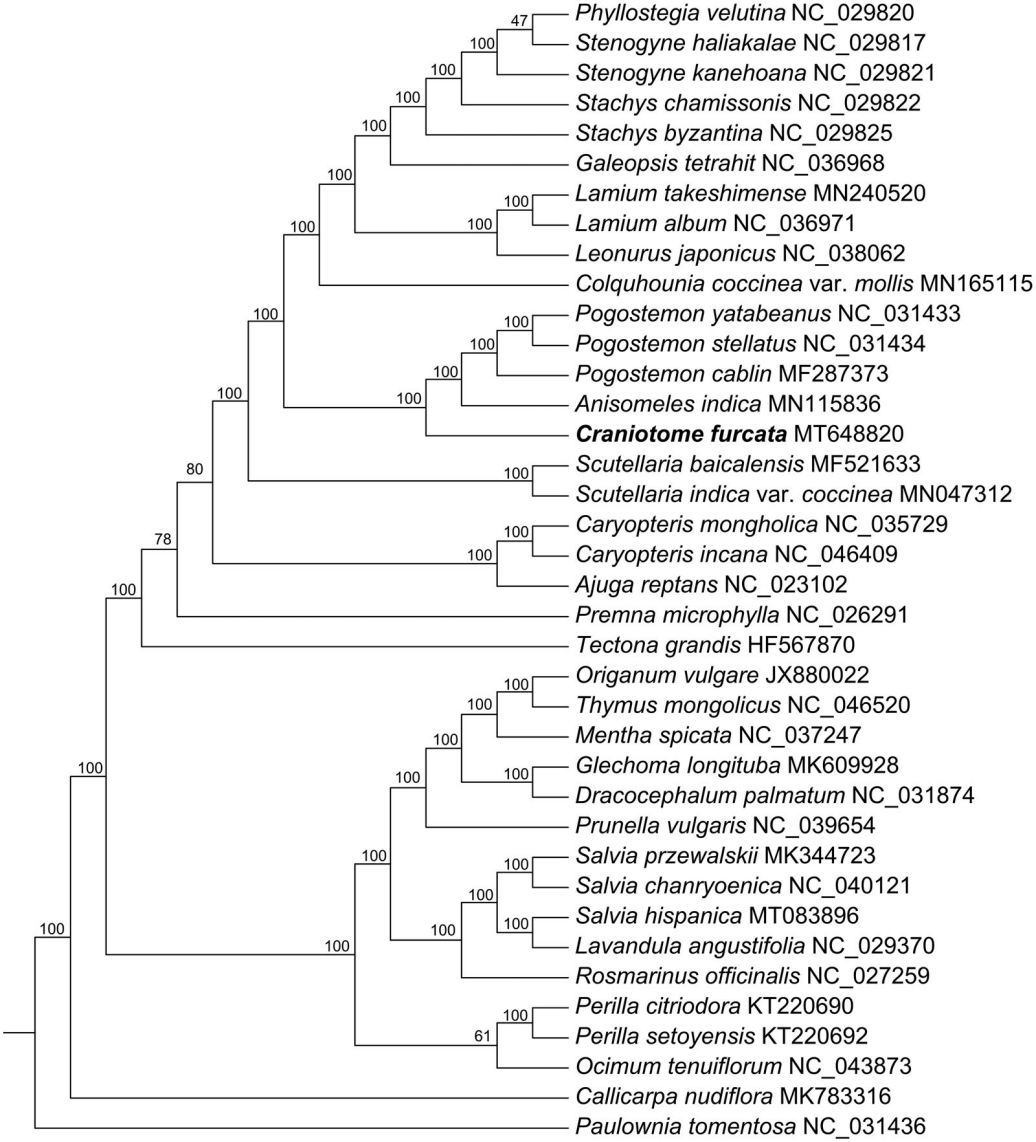
Maximum-likelihood tree of Lamiaceae inferred from 79 protein-coding genes of 38 plastomes (including outgroup). Bootstrap values are indicated above branches.

## Data Availability

The data that support the findings of this study is openly available in GenBank of NCBI at https://www.ncbi.nlm.nih.gov, reference number is MT648820.
